# The Effect of Phylogenetically Different Bacteria on the Fitness of *Pseudomonas fluorescens* in Sand Microcosms

**DOI:** 10.1371/journal.pone.0119838

**Published:** 2015-03-16

**Authors:** Olaf Tyc, Alexandra B. Wolf, Paolina Garbeva

**Affiliations:** Department of Microbial Ecology, Netherlands Institute of Ecology (NIOO-KNAW), PO Box 50, 6700 AB, Wageningen, the Netherlands; University of the West of England, UNITED KINGDOM

## Abstract

In most environments many microorganisms live in close vicinity and can interact in various ways. Recent studies suggest that bacteria are able to sense and respond to the presence of neighbouring bacteria in the environment and alter their response accordingly. This ability might be an important strategy in complex habitats such as soils, with great implications for shaping the microbial community structure. Here, we used a sand microcosm approach to investigate how *Pseudomonas fluorescens* Pf0-1 responds to the presence of monocultures or mixtures of two phylogenetically different bacteria, a Gram-negative (*Pedobacter* sp. V48) and a Gram-positive (*Bacillus* sp. V102) under two nutrient conditions. Results revealed that under both nutrient poor and nutrient rich conditions confrontation with the Gram-positive *Bacillus* sp. V102 strain led to significant lower cell numbers of *Pseudomonas fluorescens* Pf0-1, whereas confrontation with the Gram-negative *Pedobacter* sp. V48 strain did not affect the growth of *Pseudomonas fluorescens* Pf0-1. However, when *Pseudomonas fluorescens* Pf0-1 was confronted with the mixture of both strains, no significant effect on the growth of *Pseudomonas fluorescens* Pf0-1 was observed. Quantitative real-time PCR data showed up-regulation of genes involved in the production of a broad-spectrum antibiotic in *Pseudomonas fluorescens* Pf0-1 when confronted with *Pedobacter* sp. V48, but not in the presence of *Bacillus* sp. V102. The results provide evidence that the performance of bacteria in soil depends strongly on the identity of neighbouring bacteria and that inter-specific interactions are an important factor in determining microbial community structure.

## Introduction

Culture-independent technologies have given us insight in the tremendous phylogenetic and functional diversity of microbial communities [1,2]. Recently, the role of interactions between the members of microbial communities and how these shape community composition and dynamics is receiving increasing interest [3–6]. Both theoretical models and empirical studies are used to explain the coexistence of competing microbial species and consequently microbial community assembly [5,7].

In soil and in the rhizosphere, many microbial species live in close vicinity and interact with each other in various ways ranging from competition to cooperation [5,8,9]. Bacteria can recognise cues from their environment to modulate behaviour in order to increase their chance of survival.

Using recently developed techniques (NanoDESI and MALDI-TOF imaging mass spectrometry) Traxler and co-authors indicated the importance of interspecific interactions for triggering the production of different secondary metabolites in a single strain [10]. Recent studies in our group also indicate that bacteria are respond differently to the presence of different microbial species [11–13]. Studies on behavior and the transcriptional responses of the soil bacterium *Pseudomonas fluorescens* Pf0-1 on nutrient-poor agar in confrontation with taxonomically different bacterial species revealed significant differences in the responses of *Pseudomonas fluorescens* Pf0-1 to different bacteria. In particular, the expression of genes involved in signal transduction and antibiotic production was strongly affected by the identity of the interacting strains [12].

So far the response of *Pseudomonas fluorescens* Pf0-1 to phylogenetically different bacteria has only been studied during one-to-one confrontations on agar media [12,13]. However, these conditions are very artificial compared to the situation in the natural soil environment, which is a heterogeneous and complex habitat consisting of aggregated particles with huge bacterial diversity [2,14,15]. It is thus plausible that bacteria can sense more easily the presence of neighbours in their vicinity on an agar plate than in soil. Furthermore, in natural environments bacteria are likely to encounter several different competitors at the same time or in sequential events [6]. In the present study, we made a first attempt to study bacterial interactions in soil-like systems. To this end we investigated the interaction between *Pseudomonas fluorescens* Pf0-1 with monocultures and mixtures of *Pedobacter* sp. V48 and *Bacillus* sp. V102 in sand microcosms under two different nutrient conditions. We hypothesised that both nutrient conditions and the identity of the competitor would have an effect on the performance *of Pseudomonas fluorescens* Pf0-1.

## Material and Methods

### Bacterial strains and growth conditions

Three different bacterial species, *Pseudomonas fluorescens* Pf0-1 (γ-Proteobacteria) [16], *Pedobacter* sp. V48 (Sphingobacteria) and *Bacillus* sp. V102 (Bacilli) [11] were used in this study (Table 1). The strains were pre-cultured from frozen −80°C glycerol stocks on 1/10^th^ TSB agar (5.0 gL^−1^ NaCl (Merck), 1.0 gL^−1^ KH_2_PO_4_; 3 gL^−1^ Tryptic Soy Broth (OXOID); 20 gL^−1^ Agar (Merck), pH 6.5) [13] for three days at 20°C.

**Table 1:** Bacterial strains and used antibiotics / selection method applied in the selective cell counting.

Bacterial strain	Description	Reference	Applied antibiotic / selection method
*P*. *fluorescens* Pf0-1	soil isolate, Gram-negative, Class Gamma-proteobacteria	Compeau *et al*., (1988)	Ampicillin (100 μg/mL)
*Pedobacter* sp. V48	soil isolate, Gram-negative, Phylum: Bacteroidetes	de Boer *et al*., (2007)	Kanamycin (50 μg/mL)
*Bacillus* sp. V102	soil isolate, Gram-positive, Class Bacilli	de Boer *et al*., (2003)	pasteurization 10 min. @ 80°C

### Microcosm setup

Microcosms were established in 100 mL glass vials with a plastic screw cap lid (S2 Fig.) containing sterile acid washed sea sand with pore size fractions ranging from 0.075 to 0.425 mm (Honeywell Specialty Chemicals Seelze GmbH, Germany). The amount of sand was either 25 g (Microcosms supplemented with 1.5 mL 1/10^th^ strength Tryptic Soy Broth (nutrient rich media) (5.0 gL^−1^ NaCl (Merck), 1.0 gL^−1^ KH_2_PO_4_; 3 gL^−1^ Tryptic Soy Broth (OXOID)) or 30 g (Microcosms supplemented with 1.5 mL nutrient poor media (5.0 gL^−1^ NaCl (Merck), 1.0 gL^−1^ KH_2_PO_4_; 0.1 gL^−^ (NH_4_)_2_SO_4_; 0.5 gL^−1^ Tryptic Soy Broth (OXOID)). The sand was weighed directly into the glass vials and afterwards sterilized by autoclaving for 20 minutes. The sterilized microcosms were dried overnight in a 60°C oven prior to inoculation. All treatments were performed in triplicates over a time of 6 days. A detailed overview of all treatments and controls is given in Table 2.

**Table 2:** Overview of the twelve microcosm treatments.

Treatment codec	Involved bacterial strains	Number of tested interactions	Supplied growth media
**1**	***P*. *fluorescens* Pf0-1, *Pedobacter* sp. V48, *Bacillus* sp. V102**	**3**	**1/10th TSB**
**2**	***P*. *fluorescens* Pf0-1, *Pedobacter* sp. V48**	**2**	**1/10th TSB**
**3**	***P*. *fluorescens* Pf0-1, *Bacillus* sp. V102**	**2**	**1/10th TSB**
**4**	***P*. *fluorescens* Pf0-1, *Pedobacter* sp. V48, *Bacillus* sp. V102**	**3**	**Nutrient poor media**
**5**	***P*. *fluorescens* Pf0-1, *Pedobacter* sp. V48**	**2**	**Nutrient poor media**
**6**	***P*. *fluorescens* Pf0-1, *Bacillus* sp. V102**	**2**	**Nutrient poor media**
	**Controls**		
**7**	***P*. *fluorescens* Pf0-1 Monoculture**	-	**1/10th TSB**
**8**	***Pedobacter* sp. V48 Monoculture**	-	**1/10th TSB**
**9**	***Bacillus* sp. V102 Monoculture**	-	**1/10th TSB**
**10**	***P*. *fluorescens* Pf0-1 Monoculture**	-	**Nutrient poor media**
**11**	***Pedobacter* sp. V48 Monoculture**	-	**Nutrient poor media**
**12**	***Bacillus* sp. V102 Monoculture**	-	**Nutrient poor media**

### Microcosm inoculation

Sand microcosms were inoculated with either each strain as monoculture, pairwise combinations, or with all three strains together (Table 2). To inoculate the microcosms a single colony of the respective strain was transferred into 10 ml of 1/10^th^ TSB and grown overnight at 20°C, 220 rpm to an optical density (OD_600_) of: ∼0.700 (*Pseudomonas fluorescens* Pf0-1), ∼0.600 (*Pedobacter* sp. V48) and ∼0.650 (*Bacillus* sp. V102).The bacterial strains were diluted in an nutrient rich or nutrient poor inoculation master mix to a density of ∼1 * 10^5^ CFU/mL. Each microcosm was inoculated with a volume of 1.5 mL of the respective inoculum master mix in the middle of the sterilized sand and mixed well.

To verify bacterial cell numbers in the inoculum, dilution plating was done in duplicates on selective agar plates (*Pseudomonas fluorescens* Pf0-1: 1/10^th^ TSBA plates supplemented with 100 μg/mL Ampicillin, *Pedobacter* sp. V48: 1/10^th^ TSBA plates supplemented with 50 μg/mL Kanamycin, *Bacillus* sp. V102: samples were pasteurized by heat treatment for 10 min. @ 80°C).

### Bacterial enumeration

The growth of the three bacterial strains in the different treatments was tracked by plate counting of all culturable cells (*Pseudomonas fluorescens* Pf0-1 and *Pedobacter* sp. V48) or by counting of spores and heat-stable cells (*Bacillus* sp. V102) (Table 1). The enumeration was performed as follows: after one and six days of incubation a sterilized stainless steel spoon was used for sampling by mingling the sand by a full clock- and one counter- clockwise turn. After mixing 1 g sand was taken from the center of each microcosm and transferred into a 15 mL Greiner tube. A volume of 10 ml 10 mM phosphate buffer (pH 6.5) was added and the tubes were shaken in a rotary shaker at 350 rpm for 30 minutes at 20°C. Subsequently, serial dilutions were prepared and plated in triplicates on selective media (antibiotics used are indicated in Table 1). For the enumeration of the *Bacillus* sp. V102, samples were pasteurized by heating the tubes to 80°C for 10 min in a pre-warmed heating block. The plates were incubated for two to four days at 20°C and the CFUs of the respective strains were determined.

### RNA extraction and quantitative real time PCR

The expression of gene cluster Pfl01_3463-3466, which is involved in the production of a broad-spectrum antibiotic [12] was quantified via quantitative real time PCR. Total RNA was extracted at day 6 from nutrient rich microcosms (1/10^th^ TSB) containing *Pseudomonas fluorescens* Pf0-1 as monoculture or in interaction as follows: the double volume (2mL) of RNA protect Bacteria Reagent (QIAGEN cat# 76506) was added to 1 g sand sample and centrifuged at 10,000 x g for 10 min (Sigma 3K-14 centrifuge, SIGMA Laborzentrifugen GmbH, Germany). The supernatant was discarded and the pellets were stored at −80°C until further analysis. Total RNA was extracted with the MO-BIO PowerSoil Total RNA Isolation Kit (MO-BIO cat# 12866-25) according to the manufacturer’s protocol. The RNA extracts were treated with the TURBO DNA free Kit from AMBION (cat# 1907) according to the manufacturer’s protocol to remove any remaining DNA. The RNA concentration and quality was checked on a NanoDrop Spectrophotometer (Isogen Life Science, IJssestein, the Netherlands). cDNA was synthesized from the extracted RNA with random hexamer primers from Invitrogen (cat# 48190-011) by using reverse transcriptase of the Fermentas RevertAid Premium First Strand cDNA Synthesis Kit (Fermentas cat#K1651) according to manufacturer’s protocol. The concentration and quality of the cDNA was determined using a NanoDrop spectrophotometer by measuring the A260/A280 ratio and samples were run on a 1.5% agarose gel in 0.5% TBE buffer to check size and integrity of the synthesized cDNA.

The selected gene cluster was targeted with primer combination 3463F835 (5’ ATTTTTACGCGGTCTACGC) and 3463R1036 (5’TGATCAGGTTGCTGTTTCAGG) [12] amplifying 202bp from gene Pfl01_3463 encoding the two branched-chain alpha-keto acid dehydrogenase E1 component. From each treatment, 50 ng cDNA was subjected to quantitative RT- PCR using SYBR Green PCR master mix (Applied Biosystem, Warrington, UK). Quantitative RT- PCR was performed on a Corbett Research Rotor- Gene 3000 thermal cycler (Westburg, Leusden, the Netherlands) with the following settings: initial cycle 95°C for 15min, followed by 40 cycles of 95°C for 15 sec, 56°C for 50 sec and 72°C for 50 sec. All analysis was performed in triplicate. Five standard curves (9.5 ng/μl, 0.95 ng/μl, 0.095 ng/μl, 0.0095 ng/μl and 0.00095 ng/μl) were established. Gene expression data was analysed with a post-hoc LSD- test and differences between the means of data of different *Pseudomonas* interactions were considered to be statistically different at p ≤ 0.05.

### Malthusian parameter

As an estimate for fitness of the *Pseudomonas fluorescens* Pf0-1 as monoculture or in competition with the two other strains was calculated by applying the Malthusian parameter (M) growth model [17,18]. The Malthusian parameter was calculated for both monocultures and mixed cultures by comparing the number *of Pseudomonas fluorescens* Pf0-1 individuals at an initial time (t_0_), N_0_, to the number of *Pseudomonas fluorescens* Pf0-1 individuals at a future time (t_N_): M = ln (N_t_/N_0_) / t.

### Statistical analysis

Statistical analyses of the cell counts were performed with IBM SPSS Statistics 20 (IBM, Somers, NY, USA) using one-way ANOVA and post-hoc TUKEY LSD test. Significant differences between the controls (monocultures of the respective bacterial strain) and the treatments are marked with an asterisk (p≤ 0.05).

## Results and Discussion

In the present study, we investigated how the interactions between phylogenetically different bacterial strains affect the growth of *Pseudomonas fluorescens* Pf0-1 in sand microcosms under two different nutrient conditions. Our interests were particularly focused on *Pseudomonas fluorescens* Pf0-1, as our previous research had shown that *Pseudomonas fluorescens* Pf0-1 responded differently (behaviour and gene expression profile) to phylogenetically different bacteria on nutrient poor agar [11].

**Fig 1:**
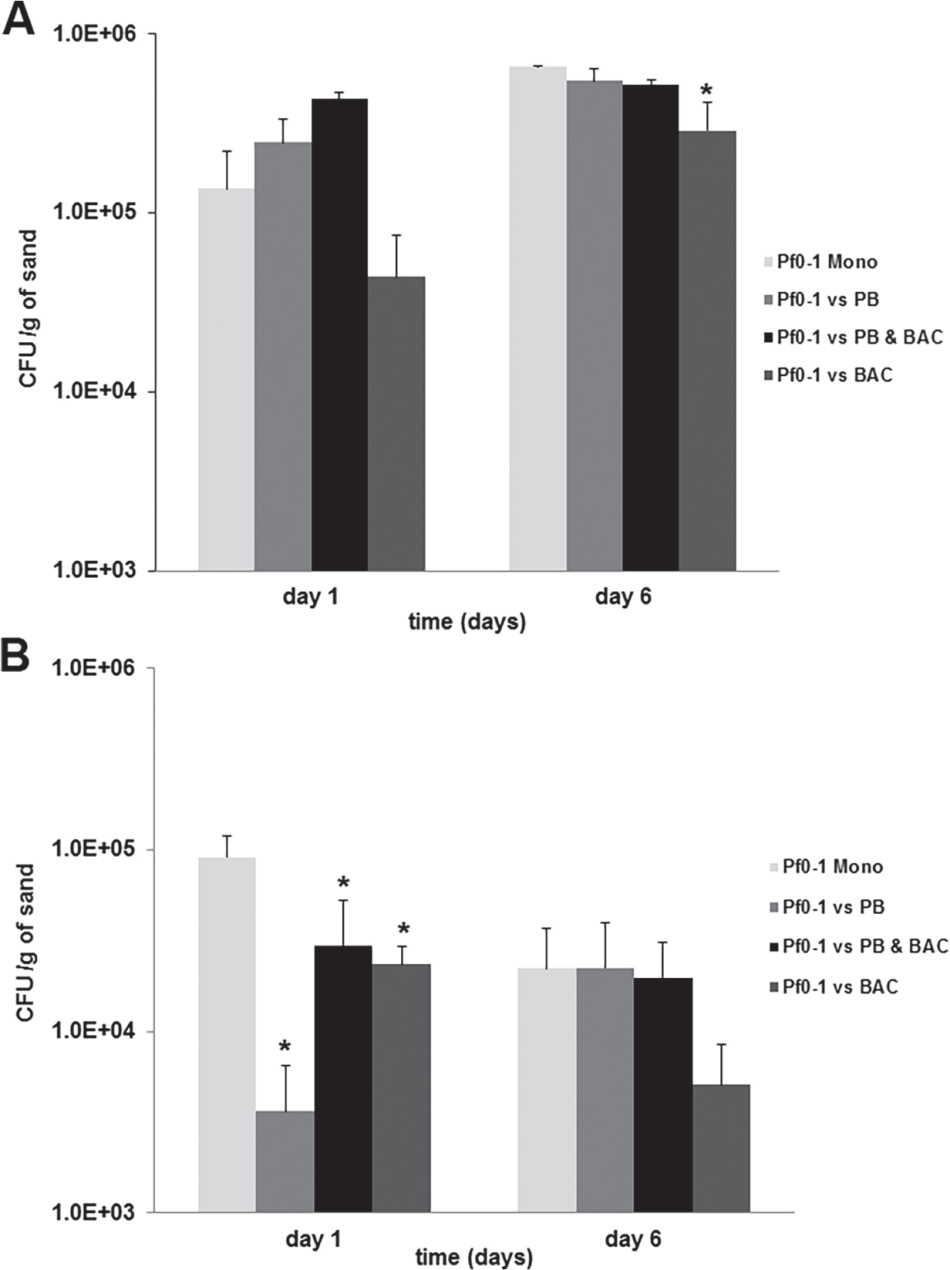
Cell counts of Pseudomonas fluorescens Pf0-1 at day 1 and day 6 under (A) nutrient rich and (B) nutrient poor conditions. Significant differences between the numbers of Pf0-1 in monoculture and in mixed cultures are indicated with an asterisk (p≤0.05). Error bars are indicating standard deviation (SD) between the triplicates. Abbreviations: Pf0-1: *Pseudomonas fluorescens* Pf0-1, PB: *Pedobacter* sp. V48, BAC: *Bacillus* sp. V102.

**Fig 2:**
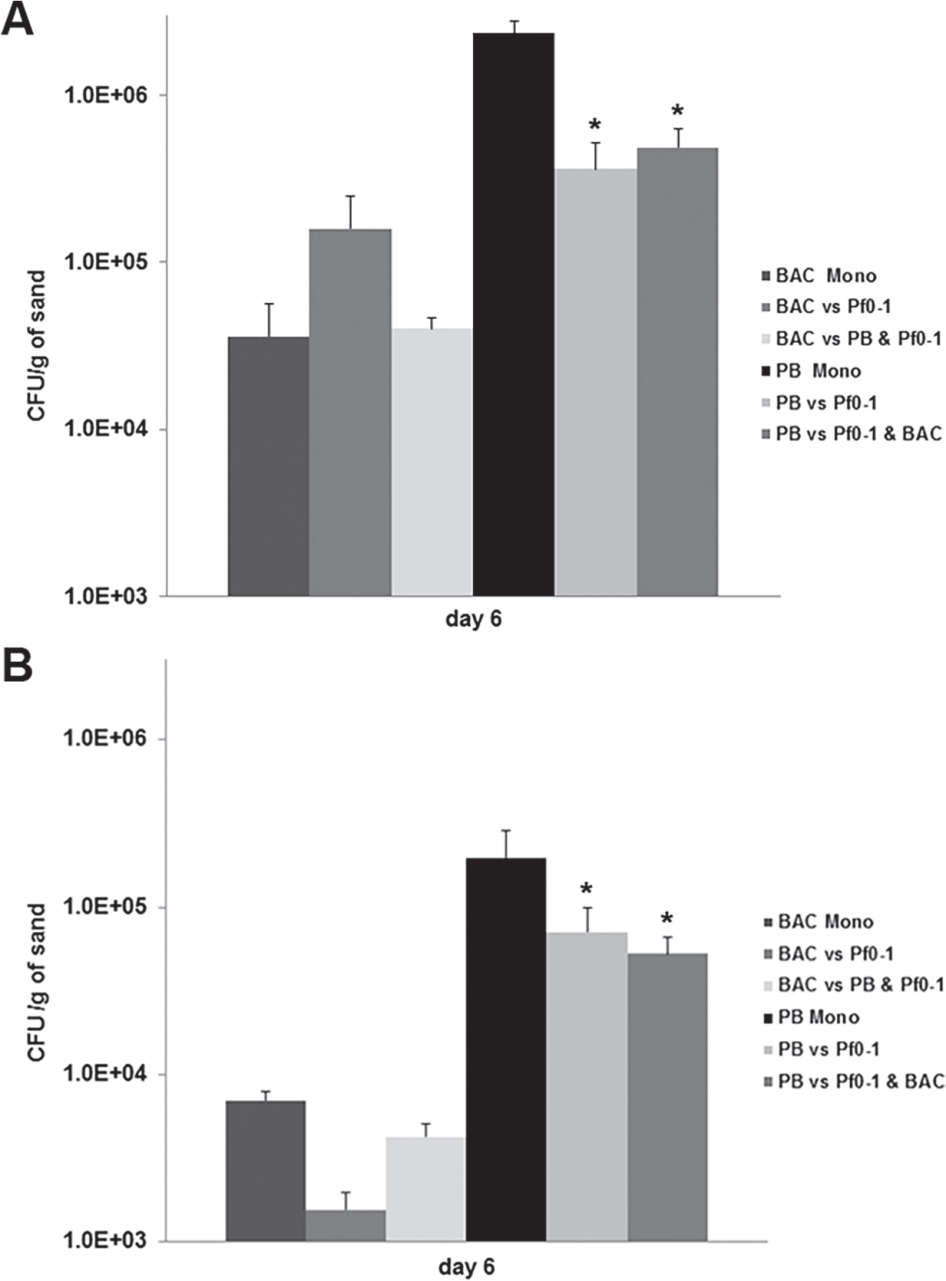
Numbers of CFUs of Bacillus sp. V102 and Pedobacter sp. V48 in monoculture and in mixed cultures (with strain Pf0-1) at day 6 under nutrient rich conditions (A) and under nutrient poor conditions (B). Significant differences between the CFUs in monoculture and in mixed cultures are indicated with an asterisk (p≤0.05). Error bars are indicating standard deviation (SD) between the triplicates. Abbreviations: Pf0-1: *Pseudomonas fluorescens* Pf0-1, BAC: *Bacillus* sp. V102, PB: *Pedobacter* sp. V48.

The growth of *Pseudomonas fluorescens* Pf0-1 in microcosms supplemented with either nutrient rich or nutrient poor growth media are presented in Fig. 1A and 1B. Bacterial enumeration revealed that all tested bacterial strains used in this study were able to grow in the sand microcosms although with lower numbers under nutrient poor conditions (Fig. 2A and B). In microcosms supplemented with nutrient rich media *Pseudomonas fluorescens* Pf0-1 reached approximately 5.5 * 10^5^ cells/g of sand as a monoculture, while in microcosms supplemented with nutrient poor media reached only 9.2 * 10^4^ cells/g of sand. The *Bacillus* sp. V102 cell numbers in monocultures reached 3.6 * 10^4^ cells/g of sand in nutrient rich microcosms and 7 * 10^3^ cells/g of sand in nutrient poor microcosms. The cell counts of *Pedobacter* sp. V48 as monoculture were approximately 2.3 * 10^6^ cells/g of sand in nutrient rich and 2.8 * 10^4^ cells/g of sand in nutrient poor microcosms (Fig. 2A and 2B).

The growth of *Pseudomonas fluorescens* Pf0-1 was negatively affected when confronted with the Gram-positive *Bacillus* sp. V102 strain resulting in significantly lower cell counts at day 6 in nutrient rich microcosms (p = 0.012) and at day 1 in nutrient poor microcosms (p = 0.008). When co-cultivated with *Bacillus* sp. V102 *Pseudomonas fluorescens* Pf0-1 reached a maximum of approximately 4.8 * 10^5^ cells/g of sand (Fig. 1A and B). Strong reduction of *Pseudomonas fluorescens* Pf0-1 growth during confrontation with *Bacillus* sp. V102 was observed previously on nutrient-poor agar even without direct cell-cell contact [12]. When co-cultivated with the Gram-negative *Pedobacter* sp. V48 strain, no significant effect on the growth of *Pseudomonas fluorescens* Pf0-1 was observed at day 6 (p = 0.988), whereas there was a significant reduction at day 1 in nutrient-poor microcosms (p = 0.000). Based on the cell enumeration we applied the Malthusian growth model (population growth) as an estimate for fitness (S1 Fig.). This revealed that the population growth of *Pseudomonas fluorescens* Pf0-1 was significantly negative affected only during co-cultivation with *Bacillus* sp. V102 on both day 1 and day 6 (p = 0.026 and p = 0.014).

**Fig 3:**
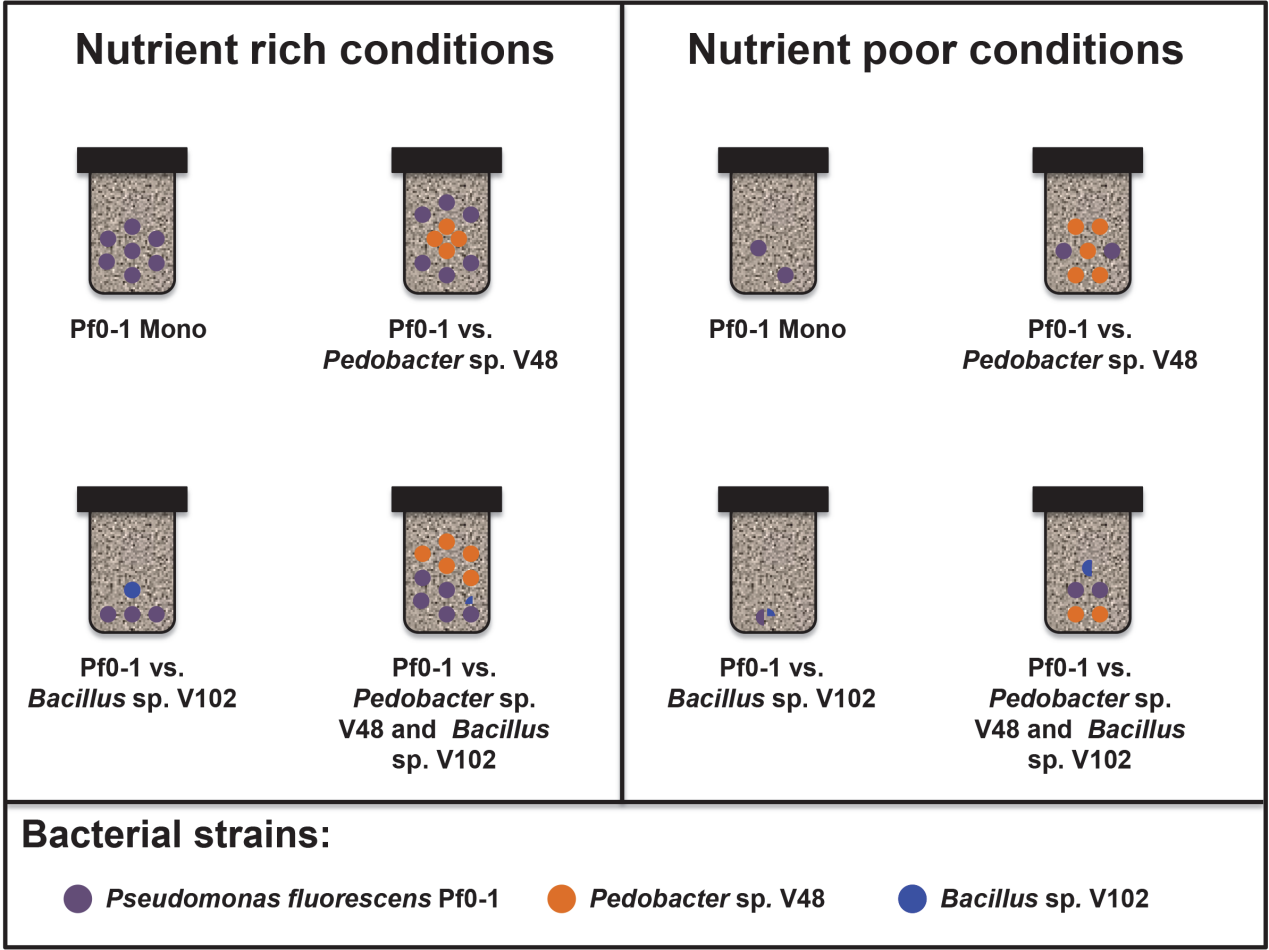
Schematic representation of the fitness of *Pseudomonas fluorescens* Pf0-1 during inter-specific interactions with either *Pedobacter* sp. V48 or *Bacillus* sp. V102 (2-way interaction) or with both strains together (3-way interaction) at day 6. The effects of the respective interaction on the fitness of *Pseudomonas fluorescens* Pf0-1 are indicated by the number of the colored circles. Each full circle represents 1.0 * 10^5^ CFU/mL (nutrient rich media) or 1.0 * 10^4^ CFU/mL (nutrient poor media).

The observed difference in response of strain *Pseudomonas fluorescens* Pf0-1 to co-cultivated bacteria was not due to the difference in bacterial growth as both *Pedobacter* sp. V48 and *Bacillus* sp. V102 were growing in the sand microcosms with *Pedobacter* sp. V48 reaching higher cell counts per gram of sand than *Bacillus* sp. V102 (Fig. 2A and 2B). However, when co-cultivated with both *Bacillus* sp. V102 and *Pedobacter* sp. V48 simultaneously, there was no significant effect on the growth of *Pseudomonas fluorescens* Pf0-1 in both nutrient rich (p = 0.650) and nutrient poor microcosms (p = 0.995) (Fig. 1A, 1B). From the inter-specific interactions investigated in the present study, it is clear that *Bacillus* sp. V102 acts as “bad” neighbour that can negatively affect the fitness of *Pseudomonas fluorescens* Pf0-1 as compared to the “good” neighbour *Pedobacter* sp. V48 that did not show any negative effect on the growth of *Pseudomonas fluorescens* Pf0-1. However, when co-cultivated with both strains simultaneously, *Pseudomonas fluorescens* Pf0-1 growth was better than when confronted only with *Bacillus* sp. V102 (Fig. 3).

**Fig 4:**
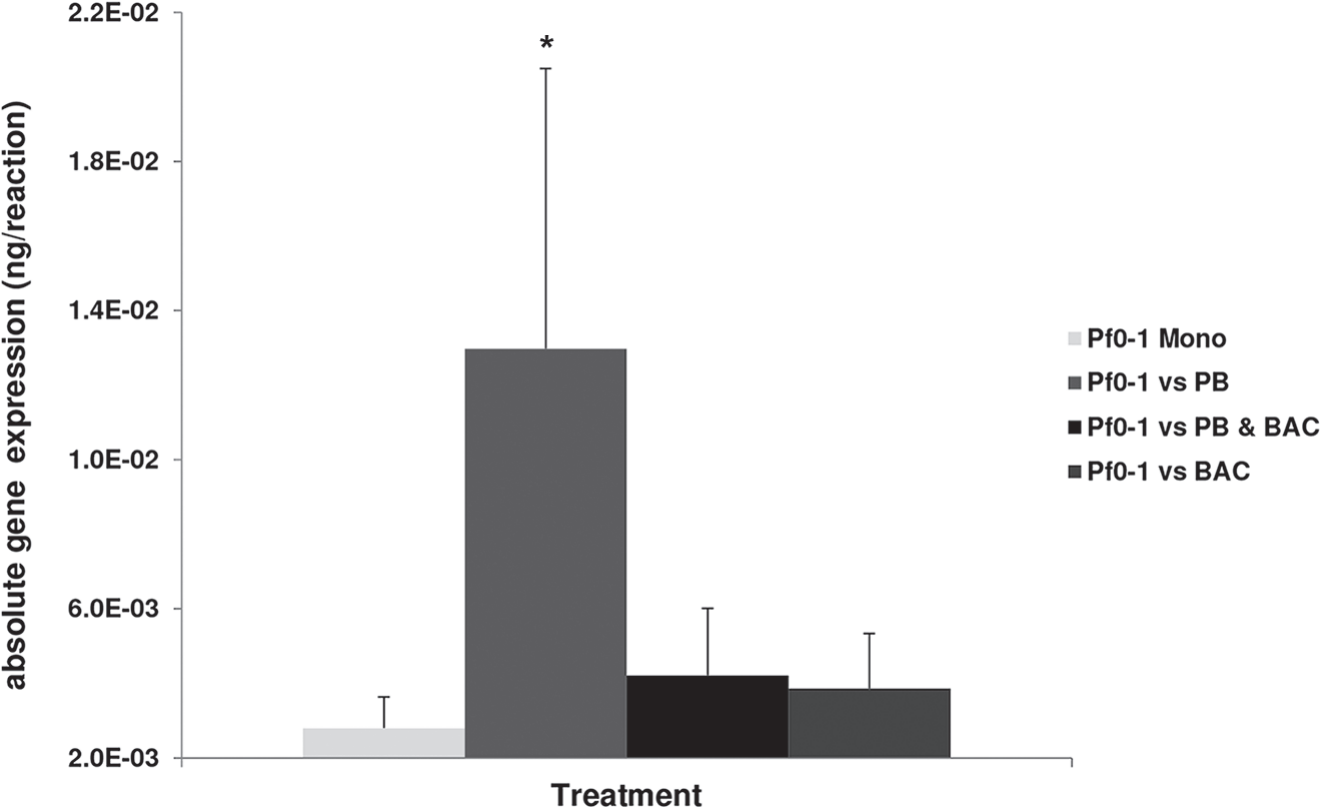
qRT-PCR results representing absolute gene expression of gene cluster Pf0-1_3463 obtained at day 6 (nutrient rich media). Error bars are indicating standard deviation (SD) between the triplicates. Significant differences between the qRT-PCR based gene expression by Pf0-1 in monocultures and mixed cultures is indicated by an asterisk (p≤0.05).

From previous studies in our group it is known that *Pseudomonas fluorescens* Pf0-1 can be triggered to produce a broad-spectrum antibiotic when co-cultivated with *Pedobacter* sp. V48, but not in co-cultivation with *Bacillus* sp. V102 [12,13]. It was hypothesized that this facultative- rather than the constitutive production of antibiotic compound represent a cost-effective strategy, as the antibiotic compound is only produced in situation when it is needed [19]. It is plausible that in a more complex habitat, the production of a broad-spectrum antibiotic triggered by *Pedobacter* sp. V48 gives *Pseudomonas fluorescens* Pf0-1a advantage when confronted with phylogenetically different strains simultaneously. To confirm that the observed results are related to antibiotic production, we performed quantitative RT-PCR with primers targeting genes Pfl01_3463 known to be involved in the production of a broad-spectrum antimicrobial compound [12]. Results revealed that indeed genes Pfl01_3463 were highly expressed at day 6 in the microcosms where *Pseudomonas fluorescens* Pf0-1 was interacting with *Pedobacter* sp. V48 (p = 0.014). Gene expression was slightly higher in treatments were *Pseudomonas fluorescens* Pf0-1 was confronted with both *Pedobacter* sp. V48 and *Bacillus* sp. V102, although not significantly (p = 0.750) (Fig. 4). Unfortunately, due to the low cell number in the microcosms supplemented with nutrient poor growth media, our attempts to extract good quality and quantity of RNA for cDNA synthesis and quantitative RT-PCR failed.

Inter-specific interactions may trigger the production of antimicrobial compounds in complex microbial communities where this so-called chemical warfare may offer comparative advantage for the producing strains [5,6]. A recent study showed that interspecific interactions between soil bacteria can have a major impact on antimicrobial compound production with effects in both directions, i.e. induction or suppression of antimicrobial compound production [20].

In soil and in the rhizosphere environment *Pseudomonas* species coexist with many other bacterial species and compete for the same nutrient resources [15,21–23]. The ability to cope with the presence of a range of competing microbial species is essential for growth and survival in soil ecosystems and the performance of soil bacteria may strongly depend on the neighbouring competitors.

Overall, our data suggests that the performance of *Pseudomonas fluorescens* Pf0-1 in sand microcosms depends greatly on the presence and identity of neighbouring microorganisms. Although *Pseudomonas fluorescens* Pf0-1 cell counts were lower in the nutrient poor sand microcosms than in the nutrient rich microcosms, similar growth patterns were observed in both experiments. This indicates that, contrary to our initial hypothesis, nutrient levels did not have a strong effect on multispecies interactions and on the ability of *Pseudomonas fluorescens* Pf0-1 to respond to different bacteria. It is well known that under different nutrient conditions bacteria often produce different secondary metabolites [24–26] and hence influence microbial interactions in different ways.

This work demonstrates that interspecific interactions can play an important role in soil and may influence microbial performance and consequently shape the composition of microbial communities.

## Supporting Information

S1 Fig Malthusian parameter calculated for the time interval from day 0 to day 1 and for the time interval from day 0 to day 6 (nutrient rich media) representing the fitness of Pseudomonas fluorescens Pf0-1 in the four different microcosm treatments. Error bars are indicating standard deviation (SD) between the triplicates. Significant differences are indicated by an asterisk (p≤0.05).
(TIF)

S2 Fig Example of a sand micorocosm used in this study. (TIF)
