# Supplementary figures and images for: The Effect of Phylogenetically Different Bacteria on the Fitness of Pseudomonas fluorescens in Sand Microcosms
Source: PLoS One. 2015 Mar 16;10(3):e0119838. doi: 10.1371/journal.pone.0119838 (PMC4361692; doi:10.1371/journal.pone.0119838)

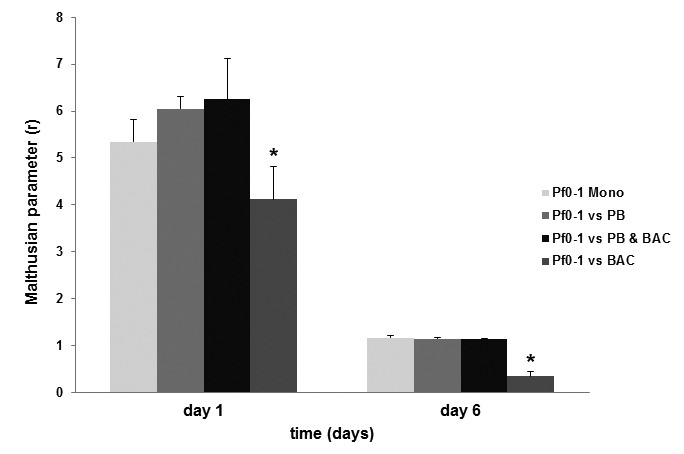

Supplement: S1 Fig — Error bars are indicating standard deviation (SD) between the triplicates. Significant differences are indicated by an asterisk (p≤0.05). (TIF) [file pone.0119838.s001.tif]

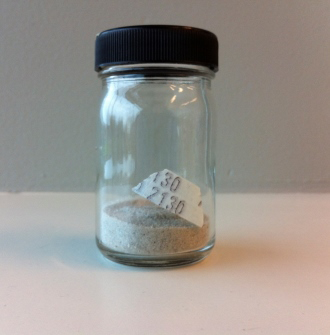

Supplement: S2 Fig — (TIF) [file pone.0119838.s002.tif]
